# 755 Utilizing a Structured Critical Care Rounding Tool for Retrospective Morbidity and Mortality Review

**DOI:** 10.1093/jbcr/irae036.297

**Published:** 2024-04-17

**Authors:** Kaitlyn M Libraro, Jamie M Heffernan

**Affiliations:** New York - Presbyterian / Weill Cornell, New York, NY; New York - Presbyterian / Weill Cornell, New York, NY

## Abstract

**Introduction:**

This 20-bed Burn ICU (BICU) previously reported on the creation and implementation of a methodical daily rounding tool for critical care burn patients. The initial goal of the tool was to improve interdisciplinary communication and investigate the characteristics of burn induced hypermetabolic response and systemic inflammatory syndrome (SIRS), a well-known and ever-challenging differential noted in the burn population. These rounding tools were completed on all critical care burn patients and ensured that necessary assessment points were evaluated the same way despite the provider on call. The quality RN then collected the tools for data collection and quality improvement review. Since its introduction, the tool has expanded its applicability from its original intention as a daily methodical rounding tool for improved interdisciplinary and MD to MD communication, to a retrospective quality tool enhancing morbidity and mortality quality reviews.

**Methods:**

The rounding sheets are completed by a resident caring for a critical care patient based on that provider’s bedside assessment and recent chart review and are then presented to the team during rounds. The rounding Attendings evaluate the information as provided and make amendments to the document as needed. Following rounds, the Attending’s completed rounding tool is provided to bedside nursing and the quality RN. During morbidity and mortality reviews, the rounding sheets are retrospectively reviewed by the quality team to evaluate potential opportunities for improvement.

**Results:**

The rounding tool has been utilized for the retrospective Quality review of 13 mortalities and 3 morbidities. The most utilized portion of the rounding tool has been the Burn Sepsis Screening Tool (BSST) section. In real time, the BSST assists in early identification of SIRS vs. hypermetabolic response. When utilized retrospectively, this tool plays a valuable role in evaluating opportunities for improvement specifically for timely diagnosis and treatment.

**Conclusions:**

Modern medicine is reliant on the use of technology and Electronic Medical Records (EMR) in recording the information of a patient’s care and management. Similarly, retrospective morbidity and mortality quality reviews are highly dependent on the data entered by the provider into the EMR. A significant limitation of the EMR is a lack of subjective provider input and clinical judgment in the time surrounding certain decision making and diagnosis. Use of the rounding tool for retrospective review has proven beneficial to this burn center’s quality review process.

**Applicability of Research to Practice:**

Directly applicable

